# Integrated analysis of inflammatory response subtype-related signature to predict clinical outcomes, immune status and drug targets in lower-grade glioma

**DOI:** 10.3389/fphar.2022.914667

**Published:** 2022-08-26

**Authors:** Yudong Cao, Hecheng Zhu, Quan Chen, Hailong Huang, Dongcheng Xie, Xuewen Li, Xingjun Jiang, Caiping Ren, Jiahui Peng

**Affiliations:** ^1^ Department of Neurosurgery, National Clinical Research Center for Geriatric Disorders, Xiangya Hospital, Central South University, Changsha, China; ^2^ Changsha Kexin Cancer Hospital, Changsha, China; ^3^ Key Laboratory for Carcinogenesis of Chinese Ministry of Health, School of Basic Medical Science, Cancer Research Institute, Central South University, Changsha, China; ^4^ Department of Ultrasound, The Seventh Affiliated Hospital, Sun Yat-sen University, Shenzhen, China

**Keywords:** lower-grade glioma, inflammatory response, immune characteristics, prognostic signature, drug targets

## Abstract

**Background:** The inflammatory response in the tumor immune microenvironment has implications for the progression and prognosis in glioma. However, few inflammatory response-related biomarkers for lower-grade glioma (LGG) prognosis and immune infiltration have been identified. We aimed to construct and identify the prognostic value of an inflammatory response-related signature, immune infiltration, and drug targets for LGG.

**Methods:** The transcriptomic and clinical data of LGG samples and 200 inflammatory response genes were obtained from public databases. The LGG samples were separated into two inflammatory response-related subtypes based on differentially expressed inflammatory response genes between LGG and normal brain tissue. Next, inflammatory response-related genes (IRRGs) were determined through a difference analysis between the aforementioned two subtypes. An inflammatory response-related prognostic model was constructed using IRRGs by using univariate Cox regression and Lasso regression analyses and validated in an external database (CGGA database). ssGSEA and ESTIMATE algorithms were conducted to evaluate immune infiltration. Additionally, we performed integrated analyses to investigate the correlation between the prognostic signature and N 6-methyladenosine mRNA status, stemness index, and drug sensitivity. We finally selected MSR1 from the prognostic signature for further experimental validation.

**Results:** A total of nine IRRGs were identified to construct the prognostic signature for LGG. LGG patients in the high-risk group presented significantly reduced overall survival than those in the low-risk group. An ROC analysis confirmed the predictive power of the prognostic model. Multivariate analyses identified the risk score as an independent predictor for the overall survival. ssGSEA revealed that the immune status was definitely disparate between two risk subgroups, and immune checkpoints such as PD-1, PD-L1, and CTLA4 were significantly expressed higher in the high-risk group. The risk score was strongly correlated with tumor stemness and m6A. The expression levels of the genes in the signature were significantly associated with the sensitivity of tumor cells to anti-tumor drugs. Finally, the knockdown of MSR1 suppressed LGG cell migration, invasion, epithelial–mesenchymal transition, and proliferation.

**Conclusion:** The study constructed a novel signature composed of nine IRRGs to predict the prognosis, potential drug targets, and impact immune infiltration status in LGG, which hold promise for screening prognostic biomarkers and guiding immunotherapy for LGG.

## Background

Gliomas are the most frequent intracranial tumors in the central nervous system (CNS) and exhibit a dismal prognosis (Ostrom et al., 2021). According to the WHO histopathological grading system, lower-grade gliomas (LGGs) include grade II and III astrocytomas, oligodendrogliomas, and oligoastrocytomas (Brat et al., 2015). Although the prognosis of LGG patients is generally good, the nature of invasiveness and malignant progression makes the treatment of LGG challenging (Yue et al., 2012). Currently, the main treatment for LGG patients consists of surgery followed by radiotherapy and adjuvant chemotherapy. However, the existing therapies for LGG are ineffective and frequently induce hypermutated recurrent neoplasms (Johnson et al., 2014), highlighting the need for a novel understanding of molecular mechanisms underlying LGG.

Inflammation is closely related to tumor progression. As early as 1863, Rudolf Welshaw first described inflammatory cells infiltrating the tumor (“lymphoreticular infiltration”) and hypothesized that the cancer was triggered by infection and chronic inflammation (Balkwill and Mantovani, 2001). In recent years, the crucial role of inflammation in the occurrence and development of tumors has become a research hotspot (Grivennikov et al., 2010; Greten and Grivennikov, 2019). Growing evidence suggests that tumor-promoting inflammation is a chief hallmark of neoplasm (Hanahan and Weinberg, 2011; Hanahan and Coussens, 2012). Systemic inflammation markers, such as C-reactive protein, neutrophil–lymphocyte ratio, and platelet–lymphocyte ratio, have been identified as pivotal parameters that can forecast the cancer prognosis in multiple cancers (Wang et al., 2016), including glioma (Lee et al., 2020). However, the levels of neutrophils, lymphocytes, and platelets in peripheral blood were insufficient to evaluate alterations in inflammatory microenvironments within a neoplasm tissue. Like other malignancies, the initial development of gliomas has also been shown to be closely associated with the inflammatory response and immune state (Michelson et al., 2016); however, the potential mechanism of the inflammatory response in glioma remains unknown.

In recent years, a series of potential diagnostic or predictive biomarkers, as well as prognostic signatures or subtypes, for glioma have been identified based on public databases, including The Cancer Genome Atlas (TCGA), Chinese Glioma Genome Atlas (CGGA), and Gene Expression Omnibus (GEO). In LGG, Ni et al. (2020) have found 25 tumor immune-related prognostic genes, which provide the basis for predicting clinical outcomes in this disease. Another research established an immune-related radiosensitivity prognostic signature that could effectively predict the prognosis of LGG patients receiving radiotherapy (Yan et al., 2022). Studies have identified immune-related subsets in diffuse glioma (Zhou et al., 2020), LGG (Yang et al., 2022), and isocitrate dehydrogenase (IDH) wild-type LGG (Wu et al., 2020), which provided potential immunotherapy targets for different types of glioma. Wang et al. (2021) found that REXO2 and RUNX1 contributed to the heterogeneity and prognosis of the IDH wild-type LGG. However, the relationship between an inflammatory response and the LGG prognosis remains largely unexplored. A comprehensive analysis was conducted to explore inflammatory response-related prognostic makers in LGG to gain an insight into the role of the inflammatory response in tumorigenesis and development.

The present study acquired the mRNA expression profiles and corresponding clinical data of the LGG patients from public databases. Then, we constructed two inflammatory response-related subtypes based on differentially expressed inflammatory response genes (DE-IRGs) between the LGG samples and normal brain tissues from the TCGA and Genotype-Tissue Expression Project (GTEx) cohorts, respectively. By using differential expression analyses in these two subtypes, univariate Cox regression and the least absolute shrinkage and selection operator (LASSO) Cox regression analysis, we finally established a prognostic signature and validated the reliability and stability of this model through the CGGA cohort. A functional enrichment analysis was also carried out to investigate its potential mechanisms. Furthermore, we also investigated the relationships between risk scores and immune characteristics. Finally, we analyzed the association of prognostic gene expression with tumor stemness, N6-methyladenosine (m6A) mRNA status, and cancer chemoresistance to bring a novel perspective on predicting the prognosis and improving treatment strategies for patients with LGG; and identified MSR1, associated with the migration, invasion, epithelial–mesenchymal transition (EMT), and proliferation of LGG cells, as a potential drug target.

## Materials and methods

### Data collection

**FIGURE 1:**
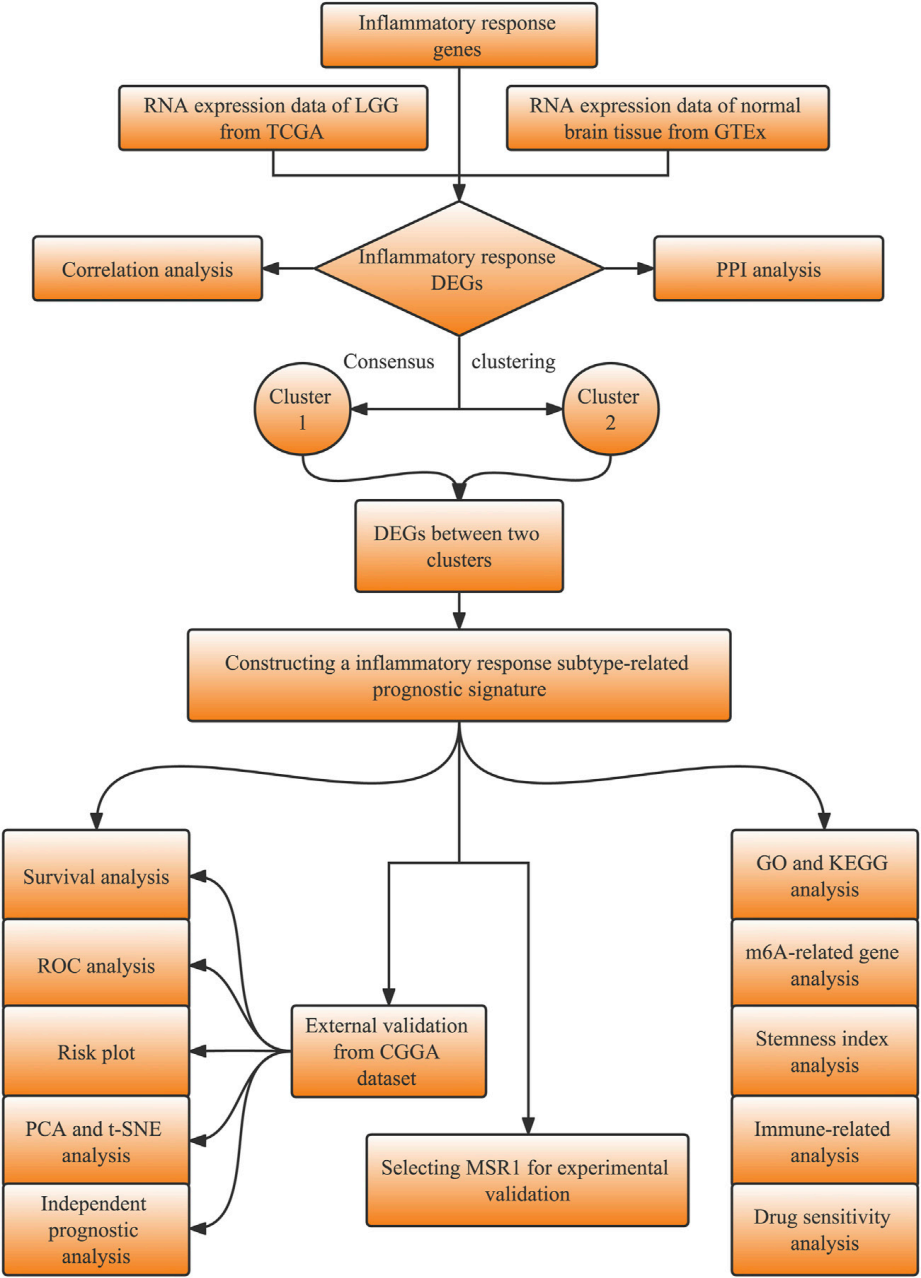
The overall procedure flow chart illustrating the data collection and analysis process.

The study design is shown in the flowchart (Figure 1). The RNA sequencing (RNA-seq) data with the format of fragments per kilobase of per million (FPKM) and the corresponding clinical information of LGG patients were obtained from the TCGA database (https://portal.gdc.cancer.gov). The RNA-seq data in normal brain tissues were downloaded from the database of the GTEx on the UCSC Xena website (https://xena.ucsc.edu/). The validation datasets, including the RNA-seq and corresponding clinical data of LGG, were downloaded from the CGGA database (http://www.cgga.org.cn/). Inflammatory response genes (IRGs, listed in Supplementary Table S1) were retrieved in the molecular signatures database (MSigDB, http://www.gsea-msigdb.org/gsea/msigdb/index.jsp).

### Identification of differentially expressed inflammatory response genes between normal and cancer samples

To compare with GTEx data, gene expression RNA-seq of LGG in TCGA was also downloaded from the UCSC Xena website. Both profiles were re-computed from raw RNA-Seq data by the UCSC Xena project and converted to the format as log2 (FPKM+1). The “normalizeBetweenArrays” function in the “limma” package of R was performed for the quantile normalization of combining the TCGA and GTEx data. The DE-IRGs were identified using the “limma” R package with a false discovery rate (FDR) < 0.05 and |log2FC| ≥ 2. A protein–protein interaction (PPI) network for the DE-IRGs was constructed from Search Tool for the Retrieval of Interacting Genes (STRING), v11.0 (https://string-db.org/). The correlation network of the DE-IRGs was displayed using “reshape2” and “igraph” R packages.

### Clustering analysis based on differentially expressed inflammatory response genes

We then performed a consensus clustering analysis with all LGG patients in the TCGA cohort based on the DE-IRGs to explore the connections between the expression of the DE-IRGs and LGG subtypes, leading to distinct molecular characteristic clinical outcomes. A consensus clustering analysis was performed using the “ConsensusClusterPlus” R package and 1,000 repetitions were performed to guarantee the stability of our classification. The highest intragroup correlations and the lowest intergroup correlations were shown with clustering variable (k) = 2 when k increased from 2 to 10. The overall survival (OS) time between the two clusters was compared with the “survival” and “survminer” R packages. The clinical characteristics, such as age, gender, grade, and molecular features including IDH1 status, 1p/19q codeletion, MGMT promoter status, and ATRX status between the two clusters, were displayed in a heatmap using the “pheatmap” R package.

### Construction and validation of an inflammatory response-subtype-related gene prognostic signature

The differentially expressed genes between the inflammatory response-related subtypes, namely, inflammatory response subtype-related genes (IRRGs), were selected by the “limma” R package with FDR <0.05 and |log2FC| ≥ 2 in the TCGA cohort. Samples with follow-up time >30 days were kept. The univariate Cox regression analysis based on IRRGs was performed via the “survival” R package with *p <* 0.001 as the criterion to select prognostic IRRGs. Based on these prognostic IRRGs, the LASSO Cox regression analysis was then utilized to narrow down the candidate genes with a tenfold cross-validation and construct the prognostic model using the “glmnet” R package. The risk scores of the patients were calculated by the formula as follows: Risk Score = $\overset{i}{\underset{1}{\sum }}Coef\left(i\right)\text{*}Exp\left(i\right)$ where Coef and Exp represented the coefficient and the expression level of every retained gene in the TCGA cohort, respectively. Using the median risk score of the TCGA cohort as the cutoff, patients in the TCGA and CGGA cohorts were sorted into high-risk and low-risk groups, respectively.

We performed a principal component analysis (PCA) and a t-distributed Stochastic Neighbor Embedding (t-SNE) analysis based on the expression levels of the genes in the prognostic signature to explore the distribution of different groups in the two cohorts with “Rtsne” and “ggplot2” R packages. The Kaplan–Meier (KM) analysis was implemented between the high- and low-risk groups via “survival” and “survminer” R packages in the train and validation cohorts. We applied time-dependent receiver operating characteristic (ROC) curves to examine the efficiency of the prognostic signature by “survival”, “survminer”, and “timeROC” R packages in the two cohorts. Furthermore, univariate and multivariate Cox regression analyses were performed in the two cohorts to explore the independence of the risk score and other clinical characteristics including grade, gender, age, IDH1 status, 1p/19q codeletion, and MGMT promoter status using the “survival” R package. A nomogram was also constructed integrating the prognostic signature to predict 1, 3, and 5-year OS of LGG patients in the TCGA cohort.

### Immune-related analysis in The Cancer Genome Atlas and the Chinese glioma genome atlas cohort

We performed a single-sample gene set enrichment analysis (ssGSEA) to calculate enrichment scores for each sample according to 29 immune data sets including immune cell types and immune-related functions obtained from Bindea's study (Bindea et al., 2013) using the “GSVA”, “limma” and “GSEABase” R packages. Scores on the immune cell and immune functions between the high- and low-risk groups were compared in the two cohorts via “limma”, “reshape2” and “ggpubr” R packages. Moreover, based on the ESTIMATE algorithm, ESTIMATE score, immune score, stromal score, and tumor purity were calculated, and the relationship between these scores and the risk score were evaluated to further explore the relationship between the signature and tumor microenvironment using “limma”, “ggplot2”, “ggpubr”, and “ggExtra” R packages. The ssGSEA enrichment scores and scores based on the ESTIMATE algorithm between the high- and low-risk groups were shown using “pheatmap” and “ggpubr” in R package. Given the importance of checkpoint inhibitor-based immunotherapies, the expression of immune checkpoints including SIGLEC15, TIGIT, CD274, HAVCR2, PDCD1, CTLA4, LAG3, and PDCD1LG2 were also compared between the high- and low-risk groups by “ggplot2” and “ggpubr” R packages.

### Functional enrichment analysis and chemotherapy sensitivity analysis

The differentially expressed genes (DEGs) between the high- and low-risk groups were filtered with FDR <0.05 and |log2FC| ≥ 1.585. Gene Ontology (GO), including the biological process (BP), cellular component (CC), and molecular function (MF) categories, and Kyoto Encyclopedia of Genes and Genomes (KEGG) analysis were conducted based on the DEGs between the high- and low-risk groups with “clusterProfiler”, “org.Hs.eg.db”, “enrichplot”, and “ggplot2” R packages.

The drug sensitivity data of NCI-60 cancer cell lines were downloaded from the CellMiner website (https://discover.nci.nih.gov/cellminer), and 218 drugs approved by the FDA were selected. The Pearson correlation analysis was implemented to explore the relationship between prognostic gene expression and drug sensitivity.

### Stemness index analysis and m6A-related gene analysis

The DNA methylation-based stemness indices (mDNAsi) and the mRNA expression-based stemness index (mRNAsi) for TCGA were acquired by machine learning from a previous research study (Malta et al., 2018), and the correlation analysis between risk score and cancer stemness index was performed using the Spearman correlation test. In addition, the expression levels of the m6A-related genes were compared between the high- and low-risk groups according to a previous study (Li et al., 2019).

### Cell culture and real-time quantitative polymerase chain reaction

Human glioma cell lines (SHG44 and HS683) used in the present study were drawn from Xiangya Medical School of Central South University (Changsha, China), maintained in a DMEM medium (Invitrogen) containing 10% fetal calf serum (Gibco) and incubated in a humidified atmosphere with 5% CO_2_ at 37°C. Small interfering RNAs (siRNAs) targeting MSR1 (Product number: siBDM0001) were purchased from Guangzhou RiboBio Co., Ltd. (Guangzhou, China). According to the manufacturer's instructions, Lipofectamine 2000 (Thermo Fisher Scientific) was used for siRNA transient transfection. Total RNA was harvested from cells using the Trizol lysis method and reverse transcribed into complementary DNA (cDNA) using the TransScript All-in-One First-Strand cDNA Synthesis SuperMix for qPCR kit (Transgen, China). According to the manufacturer's protocol, qRT-PCR was performed using the SYBR Green Master Mix (Vazyme) to detect the mRNA expression levels. The 2^−△△Ct^ method was used to calculate relative gene expression levels. The primers were synthesized and designed by Sangon Biotech (Shanghai, China) and their detailed sequences are given below: MSR1, Forward, 5′-GCA​GTG​GGA​TCA​CTT​TCA​CAA-3′, Reverse, 5′-AGC​TGT​CAT​TGA​GCG​AGC​ATC-3'; β-actin, forward, 5′-CAT​GTA​CGT​TGC​TAT​CCA​GGC-3′, Reverse, 5′-CTC​CTT​AAT​GTC​ACG​CAC​GAT-3'. The experiments were repeated thrice with β-actin as the housekeeping control gene.

### Transwell migration and invasion assays

Transwell migration and invasion assays were conducted as previously described (Tang et al., 2014). Each experiment was replicated thrice with three wells per group per assay.

### Western blot

The Western blot assay was performed as described previously. In short, 50 μɡ of proteins were run on a 10% SDS-PAGE gel and then transferred onto Polyvinyldifluoride (PVDF) membranes (Millipore). 5% non-fat milk in a TBST buffer was used for 1 h to block the membranes. After that, the membranes were incubated with primary antibodies at 4°C overnight, and then with secondary antibodies at room temperature for 60 min. Primary antibodies included mouse anti-beta actin (cat. no. ab8226; dilution, 1:1,000; Abcam), rabbit anti-ZO-1 (cat. no. 21773-1-AP; dilution, 1:2,000; Proteintech), rabbit anti-vimentin (cat. no. 10366-1-AP; dilution, 1:2,000; Proteintech). Secondary antibodies consisted of horseradish peroxidase (HRP)-conjugated goat anti-rabbit and goat anti-mouse IgG (dilution, 1:10,000; Sigma). Blots were developed with a chemiluminescent HRP substrate (Cat. no. WBKLS0500; Millipore) and visualized in a Bio-Rad Universal Hood II machine.

### Cell proliferation assay

Cell proliferation was determined by the Cell Counting Kit-8 (CCK-8) assay according to the manufacturer's protocol. Briefly, we plated cells at a concentration of 1 × 10^3^ cells per well in 96-well plates following transfection for 48 h. At each observed time point, the medium in the well was removed and replaced with 100 μl CCK8 solution (10 µl CCK8:100 µl culture medium). After incubation for 2 hours at 37°C, we measured the optical density (OD) of the supernatant in each well at wavelengths of 450 nm.

### Arsenic trioxide sensitivity assay

The sensitivity of glioma cells (U251 and T98G) to arsenic trioxide was measured with CCK-8 assay and 5-Ethynyl-2′-deoxyuridine (EdU) staining. For the CCK-8 assay, after 24 h of transfection, the transfected cells were seeded in 96-well plates (5 × 10^3^ cells per well) and then incubated overnight to enable the attachment. The medium without glioma cells was used as a blank control. Then, the transfected cells were treated with arsenic trioxide at a concentration of 4 μM for 24, 48, and 72 h. Lastly, the cell viability was assessed using the CCK-8 assay described previously. This formula was used to calculate cell viability: Cell viability = (OD_t_—OD_t-blank_)/(OD_0h_—OD_0h-blank_). As for the EdU staining, after 24 h of transfection, the transfected cells (5 × 10^4^ cells per well) were cultured in 96-well plates overnight. Following the attachment of the cells, they were cultured with arsenic trioxide at a concentration of 4 μM for 48 h. After that, the cells were incubated with an EdU solution at a concentration of 50 µM for 3 hours at 37°C. Next, we followed the standard staining procedure (Product number: C10310-3, Guangzhou RiboBio Co., Ltd.). Cell nuclei were stained with Hoechst33342 (Guangzhou RiboBio Co., Ltd.). The percentage of EdU-positive cells was calculated by EdU-positive cell count/Hoechst-stained cell count × 100%.

### Statistical analysis

All statistical analyses were accomplished by using R version 4.0.2 (https://www.r-project.org). The correlation was assessed by using Pearson correlation analysis. OS was detected by using the KM method and evaluated by the log-rank test. Cox regression analysis was used for determining survival status. The two groups were compared using a student's t-test. Two-tailed *p* < 0.05 was considered statistically significant.

## Results

### Identification of differentially expressed inflammatory response genes and classification of LGG patients in the Cancer Genome Atlas cohort

**FIGURE 2:**
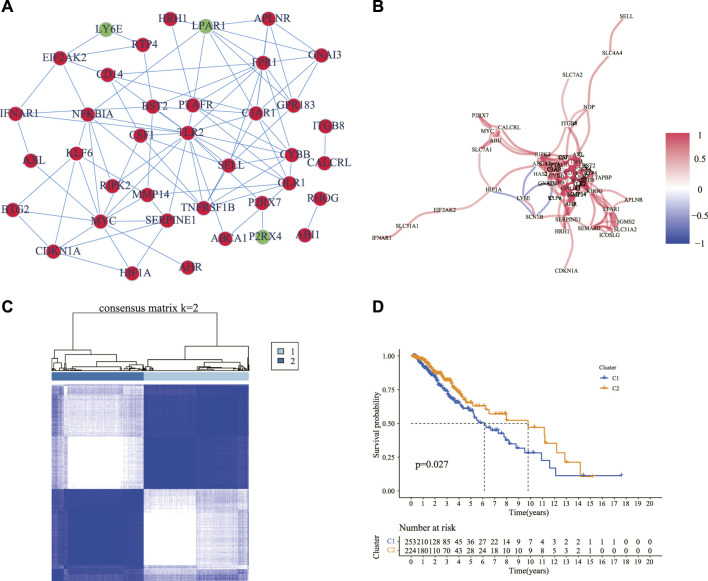
Identification of the DE-IRGs and sub-clusters based on these genes. **(A)** A PPI network showing the interactions of the DE-IRGs (interaction score = 0.9). Circles in red denote upregulated genes, and green symbolizes downregulated genes in the LGG samples compared with normal brain tissues. **(B)** The correlation network of the DE-IRGs. The red line showed a positive correlation, and the blue line showed a negative correlation. The depth of the color reflects the strength of the relevance. **(C)** Patients with LGG were classified into two clusters according to the consensus clustering matrix (*k* = 2). **(D)** Kaplan–Meier curves for the OS of patients between the two clusters. DE-IRGs: differentially expressed inflammatory response genes; PPI: protein–protein interaction; LGG: lower-grade glioma; OS: overall survival.

We explored the expression of the 200 IRGs between 529 LGG samples and 1,152 normal brain samples, and 54 IRGs were differentially expressed, with 46 upregulated and 8 downregulated in LGG samples. The PPI analysis detected the interactions of these DE-IRGs with an interaction score set at 0.9 and revealed that C3AR1, FPR1, LPAR1, GPR183, APLNR, GNAI3, TLR2, and CYBB were hub genes (Figure 2A). The correlation network of these DE-IRGs is showed in Figure 2B.

The consensus clustering analysis was performed according to the 54 DE-IRGs in the LGG samples to investigate the connections between the expression of the DE-IRGs and LGG subtypes. The number of clusters was represented by the letter “k” and consensus matrices showed that the intergroup correlations were the highest and the intragroup correlations were low with *k* = 2 (Figure 2C). It was observed that cluster 1 had a significantly poorer OS than cluster 2 (Figure 2D). Moreover, we compared the clinical manifestations including age, gender, grade, and molecular characteristics such as IDH1 status, 1p/19q codeletion, MGMT promoter status, and ATRX status between the two clusters. It was observed that patients with wild-type IDH1, non-codel 1p/19q, unmethylated MGMT promoter, and mutant ATRX status were statistically prominent in cluster 1 (Supplementary Figure S1).

### Development and validation of a prognostic signature in The Cancer Genome Atlas and Chinese glioma genome atlas cohorts

**FIGURE 3:**
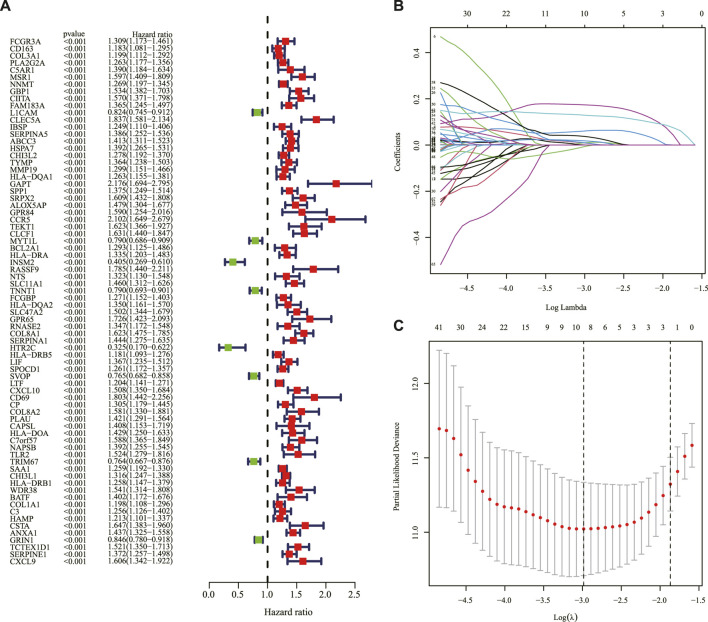
Construction of the prognostic IRRG signature. **(A)** Univariate Cox regression analysis of OS based on IRRGs in the TCGA cohort. **(B)** LASSO regression of the 74 OS-related IRRGs. **(C)** Cross-validation for tuning the parameter in the LASSO regression. IRRG: inflammatory response subtypes-related gene; OS: overall survival; TCGA: The Cancer Genome Atlas; LASSO: the least absolute shrinkage and selection operator.

The genes between the two clusters were compared, and 183 DEGs were identified with 71 genes upregulated and 112 genes downregulated in cluster 2 compared to cluster 1 in the TCGA cohort (Supplementary Table S2). The univariate Cox regression analysis was applied to screen survival-related genes based on the 183 DEGs, and 74 of them were correlated with OS (Figure 3A). After the LASSO Cox regression analysis, nine genes were selected based on the optimum λ value and constructed as a prognostic signature (Figures 3B, C). The risk score was calculated as follows: risk score = (0.0082 × expression of PLA2G2A) + (0.0105 × expression of MSR1) + (0.0611× expression of ABCC3) + (0.1737× expression of COL8A1)—(0.0052 × expression of SVOP) + (0.0091× expression of CXCL10) + (0.0365× expression of NAPSB) + (0.0178× expression of SAA1) + (0.1288 × expression of CHI3L1).

**FIGURE 4:**
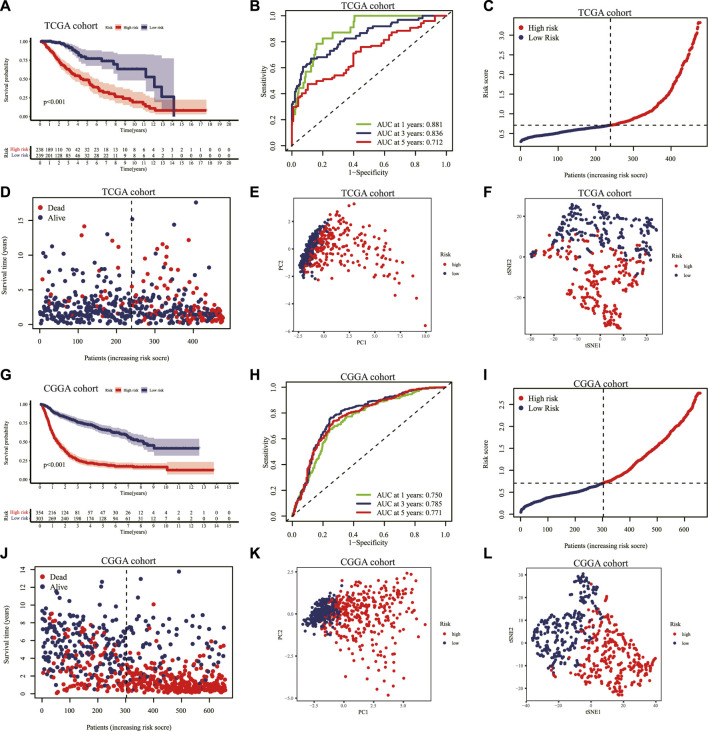
Validation of the prognostic IRRG signature. **(A)** KM curves for the OS of patients with LGG in the high- and low-risk groups in the TCGA cohort. **(B)** The ROC curve at 1-, 3-, 5-years for survival prediction of the signature in the TCGA cohort. Distribution of the risk score **(C)** and survival status **(D)** for each patient in the TCGA cohort. PCA plot **(E)** and t-SNE analysis **(F)** based on the nine prognostic signature genes in the TCGA cohort. **(G)** Similarly. KM curves for the OS of each patient in the CGGA cohort. **(H)** The ROC curve analysis of the risk score signature in the CGGA cohort. Distribution of the risk score **(I)** and survival status **(J)** for each patient with LGG in the CGGA cohort. PCA plot **(K)** and t-SNE analysis **(L)** based on the nine prognostic signature genes in the CGGA cohort. IRRG: inflammatory response-related gene; KM: Kaplan–Meier; OS: overall survival; LGG: lower-grade glioma; TCGA: The Cancer Genome Atlas; ROC: receiver operating characteristic; PCA: principal component analysis; t-SNE: T-distributed Stochastic Neighbor Embedding; CGGA: Chinese Glioma Genome Atlas.

Based on the median score in the TCGA cohort as the threshold, the LGG patients in the TCGA and CGGA cohorts were divided into low-risk and high-risk groups separately. The KM curve indicated that a shorter survival time or lower survival probability of patients was shown in the high-risk group compared to the low-risk group with 1, 3, and 5-year AUC values of 0.881, 0.836, and 0.712, respectively, in the TCGA cohort (Figures 4A, B). The distribution of risk scores and survival status of the LGG patients was also illustrated. The LGG patients’ risk of death increased, and survival probability decreased along with the risk score rising in the TCGA cohort (Figures 4C, D). The PCA and t-SNE analyses showed that LGG patients in the high- and low-risk groups were significantly separated into two clusters according to the expression of the nine prognostic genes in the TCGA dataset (Figures 4E,F).

Similarly, we validated the risk prediction formula in the CGGA cohort and identified that LGG patients with high-risk scores had a shorter survival time (Figure 4G). The ROC curve analysis displayed that the AUC values corresponding to 1, 3 and 5-year survival times were 0.750, 0.785, and 0.771 in the CGGA cohort (Figure 4H). With increasing risk scores, the LGG patients' survival time decreased, and the number of deaths escalated (Figures 4I, J). Additionally, the PCA and t-SNE analyses performed on the CGGA cohort confirmed that the low-risk and high-risk groups' patients were distributed in different directions (Figures 4K, L).

### Independent prognostic value of the signature

**FIGURE 5:**
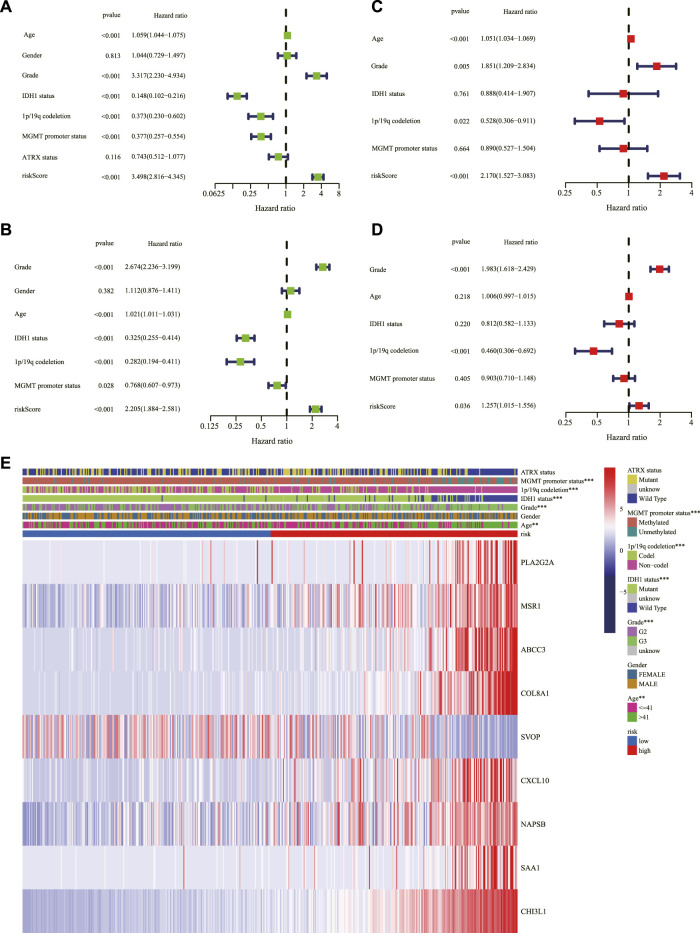
Assessment of the risk scores and the predictive value of clinical variables. Forest charts of the risk scores combining common clinical variables based on the univariate Cox regression analysis in the TCGA cohort **(A)** and the CGGA cohort **(B)**. Forest charts of the risk scores combining common clinical variables based on the multivariate Cox regression analysis in the TCGA cohort **(C)** and the CGGA cohort **(D)** showed the significance and HR values of risk scores and clinical characters. **(E)** Heatmap presented the association of risk and clinical information based on the nine-gene signature. **p < 0.01, ***p < 0.001. TCGA: The Cancer Genome Atlas; CGGA: Chinese Glioma Genome Atlas; HR: hazard ratio.

**FIGURE 6:**
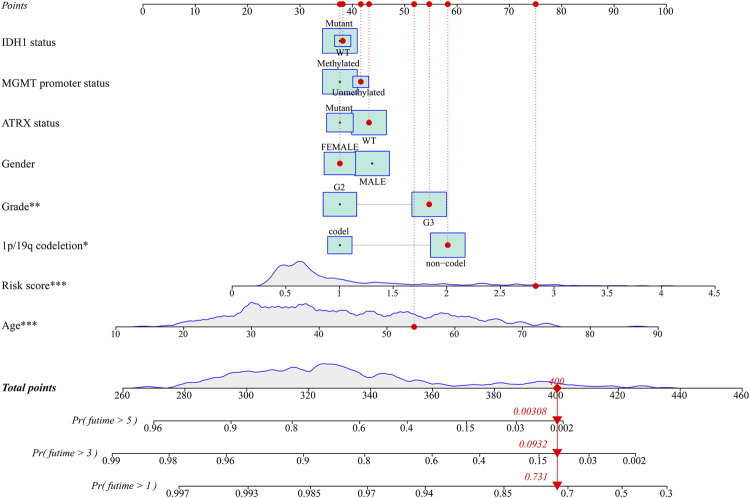
The nomogram for predicting 1-, 3-, and 5-year survival outcomes of LGG patients integrating prognostic markers including grade, gender, age, IDH1 status, 1p/19q codeletion, ATRX status, and MGMT promoter status in the TCGA cohort. LGG: lower-grade glioma; TCGA: The Cancer Genome Atlas.

Univariate and multivariable Cox regression analyses were performed in the TCGA and CGGA cohorts to evaluate whether the risk score could serve as an independent prognostic factor. The univariate Cox regression analysis revealed that risk score, age, grade, IDH1 status, 1p/19q codeletion, and MGMT promoter status could be taken as prognostic factors in the TCGA (Figure 5A) and CGGA cohorts (Figure 5B). The multivariable Cox regression analysis revealed that risk score, as well as age, grade, and 1p/19q codeletion, could be taken as independent prognostic factors in the TCGA cohort (Figure 5C), and risk score, as well as grade and 1p/19q codeletion, could be considered independent prognostic factors in the CGGA cohort (Figure 5D). In addition, the relationship between the expression of genes in the signature and clinical characteristics is displayed in Figure 5E. The nomogram presented the contribution of each influencing factor and revealed that the risk score was the leading factor for predicting OS compared to other factors (Figure 6).

### Immune status and tumor microenvironment analysis

**FIGURE 7:**
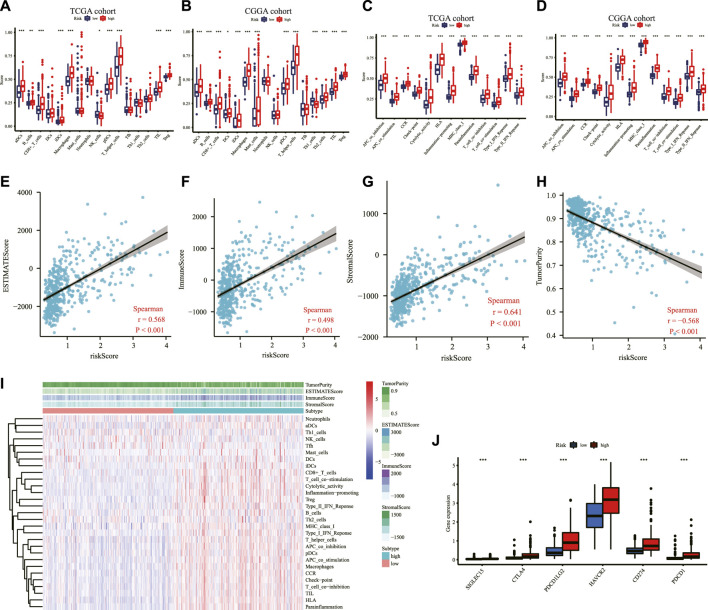
Immune-related analysis in the TCGA and CGGA cohorts. Comparison of the ssGSEA scores of immune cells between low- and high-risk groups in the TCGA cohort **(A)** and the CGGA cohort **(B)**. Comparison of the ssGSEA scores of immune-related pathways between low- and high-risk groups in the TCGA dataset **(C)** and the CGGA dataset **(D)**. The relationship between the risk score and ESTIMATE score **(E)**, immune score **(F)**, stromal score **(G)**, and tumor purity **(H)** in the TCGA cohort **(I)** Heatmap of the ssGSEA scores integrating the tumor purity, ESTIMATE score, immune score, and stromal score of each sample calculated by ESTIMATE's algorithm between the high- and low-risk groups in the TCGA dataset. **(J)** Comparison of the expression level of immune checkpoints among high- and low-risk groups in the TCGA dataset. *p < 0.05, **p < 0.01, ***p < 0.001. TCGA: The Cancer Genome Atlas; CGGA: Chinese Glioma Genome Atlas; ssGSEA: single-sample gene set enrichment analysis; ESTIMATE: Estimation of Stromal and Immune cells in Malignant Tumor tissues using Expression data.

**FIGURE 8:**
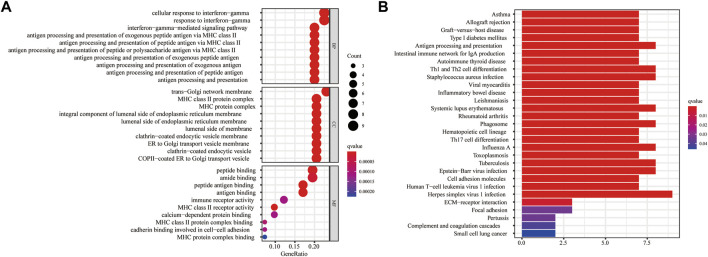
Functional enrichment analyses based on the DEGs between the high- and low-risk groups in the TCGA cohort. **(A)** The bubble graph for GO enrichment presented the top 10 terms in BP, CC, and MF. Circle size corresponded to enriched counts of genes, and the circle indicated the q-values and the significance of the enriched GO terms. **(B)** The bar plot for the KEGG pathway analysis. The bar length corresponded to the number of enriched genes in the corresponding pathway. The gradual color indicated the different degrees of KEGG enrichment, with red representing the highest magnitude of KEGG enrichment. DEG: differentially expressed gene; TCGA: The Cancer Genome Atlas; GO: Gene Ontology; BP: Biological Process; CC: Cellular Component; MF: Molecular Function; KEGG: Kyoto Encyclopedia of Genes and Genomes.

To explore the correlation between risk score and immune status, the enrichment scores of 16 types of immune cells and 13 types of immune-related pathways based on ssGSEA were compared between the high- and low-risk groups in the TCGA and CGGA cohorts. Among the immune cell terms, aDCs, B cells, CD8^+^ T cells, iDCs, macrophages, pDCs, T helper cells, TIL, and Treg were generally and significantly of high abundance in the high-risk group than in the low-risk group; in contrast, the abundance of NK cells was significantly higher in the low-risk group than in the high-risk group in the TCGA cohort (Figure 7A). Similarly, as depicted in Figure 7B, the abundance of aDCs, B cells, CD8^+^ T cells, DCs, iDCs, macrophages, mast cells, pDCs, T helper cells, Th2 cells, TIL, and Treg were significantly higher in the high-risk group while Th1 cells were expressed significantly higher in the low-risk group in the CGGA cohort. Additionally, all the 13 immune-related pathways were significantly upregulated in the high-risk group in the two cohorts (Figures 7C, D). In the analysis of the immune microenvironment, the correlation between the risk score and immune infiltration revealed that the ESTIMATE score, immune score, and stromal score were positively relative to the risk score in the TCGA cohort, and tumor purity was negatively relative to the risk score (Figures 8E–H). The ssGSEA enrichment scores and scores based on the ESTIMATE algorithm between the high- and low-risk groups are shown in the heatmap (Figure 7I). Moreover, the expression of immune checkpoints including SIGLEC15, CD274, HAVCR2, PDCD1, CTLA4, and PDCD1LG2 were all higher in the high-risk group than in the low-risk group in the TCGA cohort (Figure 7J).

### Biological function and cancer cell sensitivity to chemotherapy

For a further study of the gene functions between the high- and low-risk groups, 43 DEGs were selected between these two groups, with 38 genes upregulated and five genes downregulated in the high-risk group (Supplementary Table S3). The GO enrichment analysis indicated that the DEGs were mainly correlated with the cellular response to interferon-gamma, trans-Golgi network membrane, and peptide binding (Figure 8A). The KEGG enrichment analysis showed that the DEGs were primarily associated with herpes simplex virus 1 infection (Figure 8B).

**FIGURE 9:**
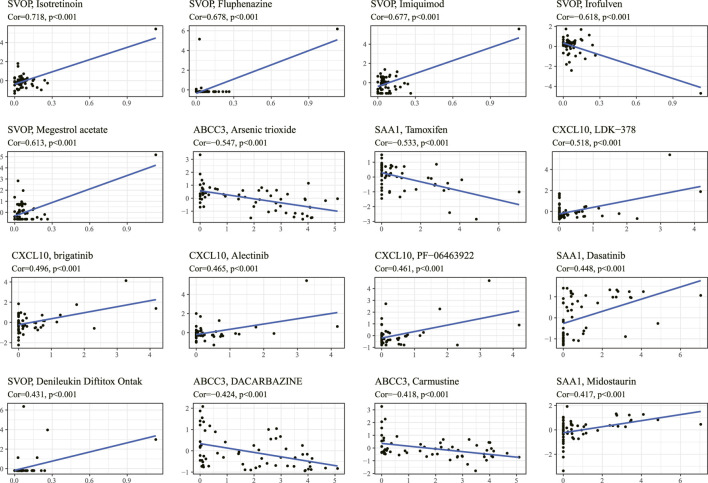
The correlation between the prognostic gene expression and drug sensitivity. The top 16 drugs with the highest correlation with gene expression in the predictive model were screened. The vertical axis shows the Z-scores of the drugs, and the horizontal axis represents the gene expression. The larger the Z-score, the more sensitive the cancer cell is to the drug.

We obtained top 16 drugs with the maximum correlation coefficient by constructing the conjunction between the expression level of prognostic IRRGs in the signature and sensitivity of chemotherapeutic agents. The results showed that the expression of SVOP was positively correlated with the sensitivity of Isotretinoin, Fluphenazine, Imiquimod, Megestrol acetate, and Denileukin Diftitox Ontak but negatively correlated with the sensitivity of Irofulven. The expression of ABCC3 was negatively correlated with the sensitivity of arsenic trioxide, DACARBAZINE, and Carmustine. In addition, the expression of SAA1 was positively correlated with the sensitivity of Dasatinib and Midostaurin but negatively correlated with the sensitivity of Tamoxifen. What's more, the higher the expression of CXCL10 in cancer cells, the stronger the cancer cells’ sensitivity to LDK-378, brigatinib, alectinib, and PF-06463922 (Figure 9).

**FIGURE 10:**
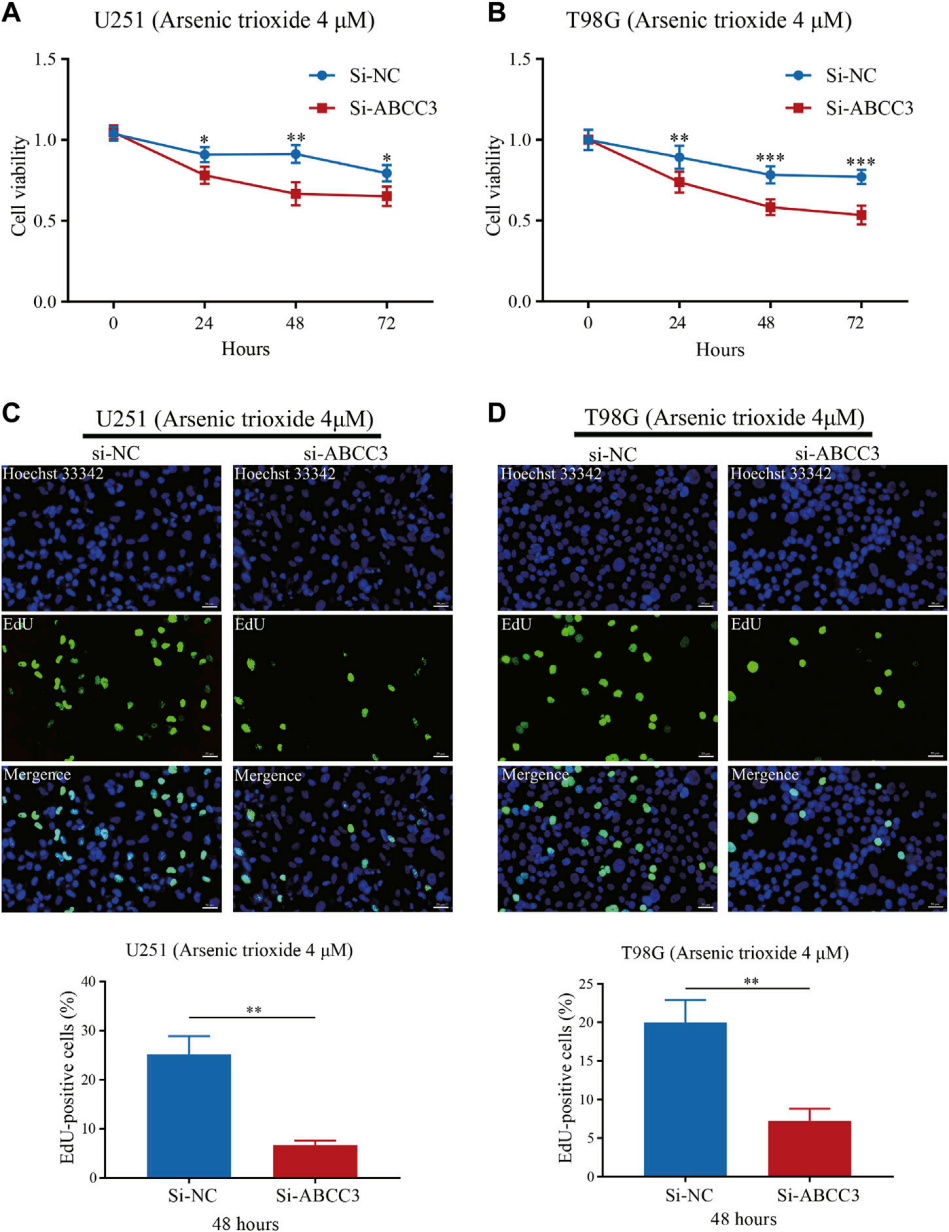
Effect of ABCC3 knockdown on the sensitivity of glioma cells to arsenic trioxide. The cell viability of U251 **(A)** and T98G cells **(B)** in the Si-ABCC3 group was significantly inhibited compared with the control group following treatment with arsenic trioxide (4 μM) for 24, 48, and 72 h. EdU staining showed that the proportion of EdU-positive cells of U251 **(C)** and T98G cells **(D)** in the Si-ABCC3 group markedly reduced compared to the control group after being treated with arsenic trioxide (4 μM) for 48 h. Photographs **(C)** and **(D)** magnification: ×200; scale bar: 50 μm. The data are presented as the mean ± SD for at least three independent experiments. *p < 0.05, **p < 0.01, ***p < 0.001. EdU: 5-Ethynyl-2′-deoxyuridine.

Next, we verified whether the knockdown of ABCC3 was associated with the increased sensitivity of glioma cells to arsenic trioxide. We treated U251 and T98G cells with arsenic trioxide at a concentration of 4 μM before the CCK-8 assay and found that the knockdown of ABCC3 markedly enhanced the repressive effect of arsenic trioxide on cell viability of U251 and T98G cells, respectively (Figures 10A,B). Likewise, the EdU assay showed a significantly lesser percentage of EdU-positive cells in the Si-ABCC3 group compared with the control group in both U251 and T98G cells after being treated with arsenic trioxide for 48 h (Figures 10C,D). The results suggested that the knockdown of ABCC3 elevated the chemosensitivity of glioma cells to arsenic trioxide.

### Correlation of the prognostic signature with the stemness index and m6A

**FIGURE 11:**
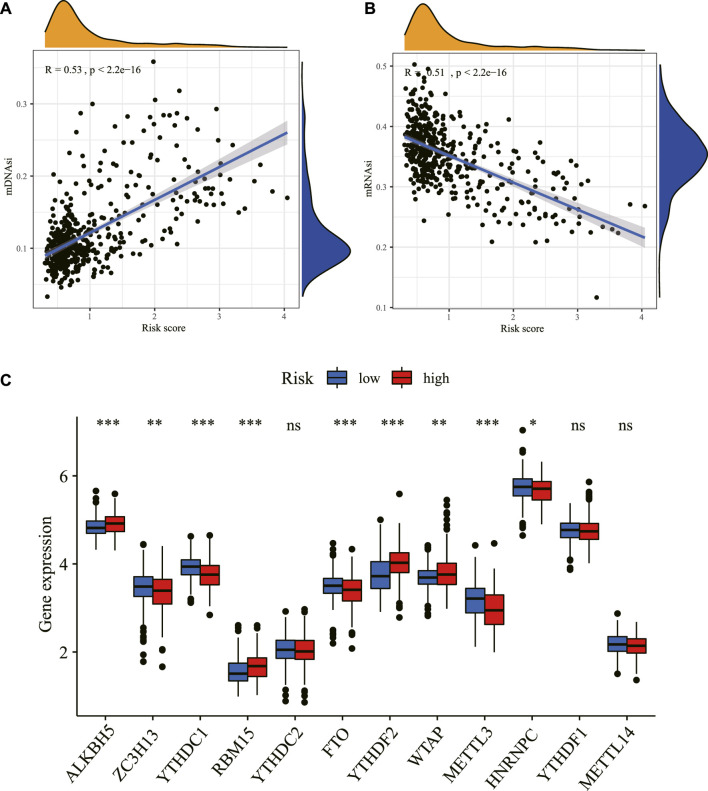
Correlation between the risk score and stemness index and m6A. The relationship between the risk score and stemness index based on mDNAsi **(A)** and the stemness index based on mRNAsi **(B)** in the TCGA dataset. **(C)** The expression level of m6A-related genes between the high- and low-risk groups in the TCGA dataset. *p < 0.05, **p < 0.01, ***p < 0.001. ns: non-sense; m6A: N6-methyladenosine; mDNAsi: DNA methylation-based stemness index; mRNAsi: mRNA expression-based stemness index; TCGA: The Cancer Genome Atlas.

The mRNAsi and the mDNAsi have been applied to assess cancer stem cell characteristics (Malta et al., 2018; Pan et al., 2019). Our results showed that the risk score was significantly positively correlated with mDNAsi while it was significantly negatively associated with mRNAsi (Figures 11A,B), consistent with a previous study (Malta et al., 2018), and demonstrated the effectiveness of the predictive model from another aspect.

Consistent with the crucial roles of m6A as a post-transcriptional gene regulatory mechanism, aberrant m6A methylation has been found to affect numerous cellular processes, including many related to tumorigenesis and tumor progression. Our study found that m6A-related mRNAs including ALKBH5, ZC3H13, YTHDC1, RBM15, FTO, YTHDF2, WTAP, METTL3, and HNRNPC were differentially expressed between high- and low-risk groups in the TCGA dataset (Figure 11C).

### Interference of MSR1 expression-debilitated glioma cell migration, invasion, epithelial–mesenchymal transition, and proliferation

Among the nine signature genes, the MSR1 gene, encoding a transmembrane protein expressed mainly by macrophages (also known as CD204), was critical to a number of physiological and pathological processes such as macrophage polarization, pathogen clearance, and lipid metabolism (Canton et al., 2013). A previous study suggested that immunoregulator CD204 could serve as an immunotherapeutic target to enhance T cell response induced by a specific dendritic cell vaccine and anti-tumor immunity (Yi et al., 2011). Thus, the MSR1 gene was chosen for further investigation in this article.

**FIGURE 12:**
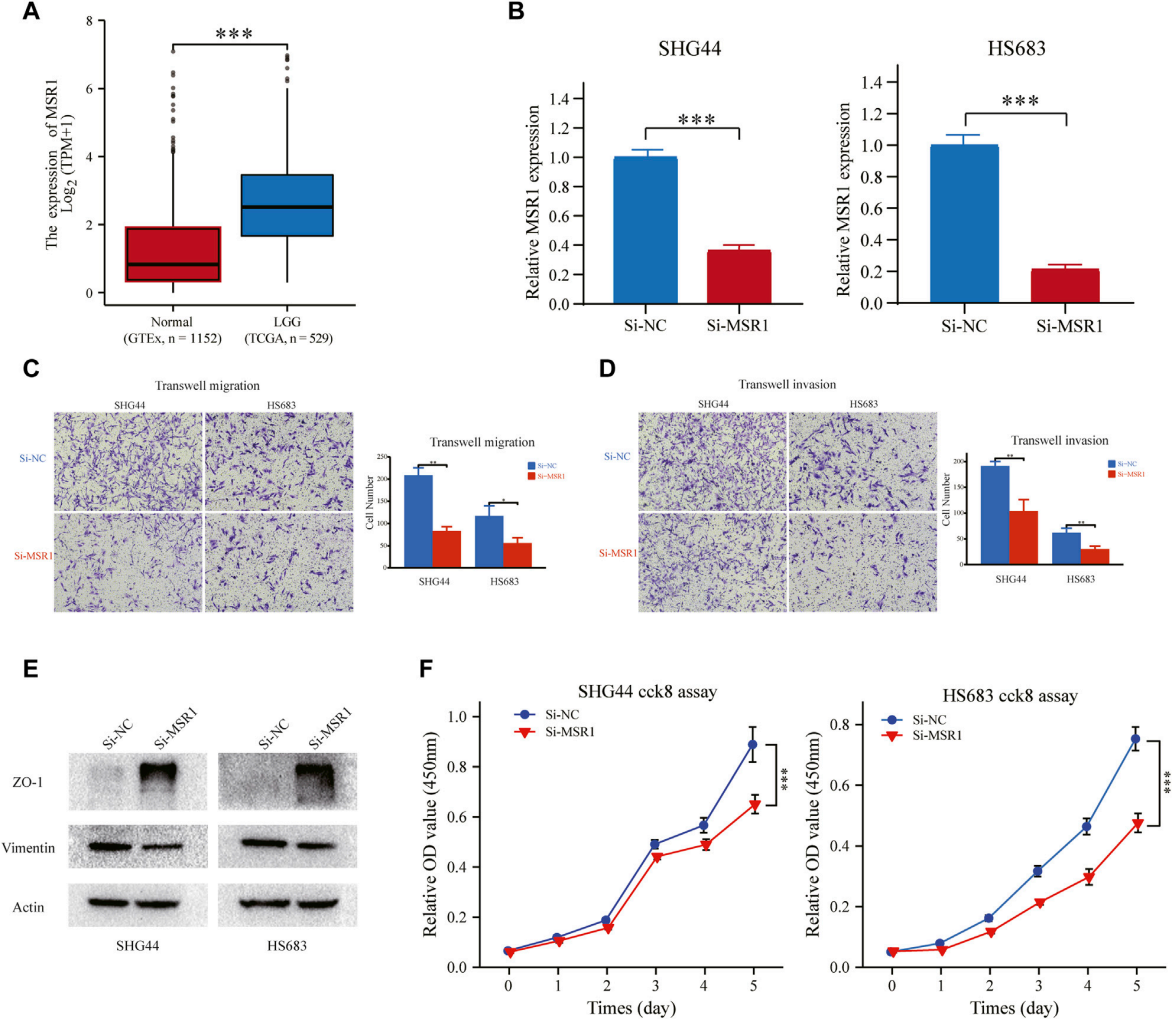
MSR1 knockdown impairs migration, invasion, EMT, and proliferation. **(A)** Relative RNA expression of MSR1 in LGG samples and normal brain tissues. **(B)** The transfection efficiency of the MSR1 siRNA in SHG44 and HS683 cells was assessed *via* qRT-PCR. **(C)** Transwell migration assay and **(D)** transwell invasion assay presented that MSR1 downregulation remarkably reduced the migration, invasion, EMT, and proliferation of SHG44 and HS683 LGG cell lines. **(E)** Western blot analysis demonstrated the expression changes in EMT markers (ZO-1 and vimentin) in the indicated LGG cell lines after MSR1 knockdown with siRNA. **(F)** CCK8 assay evaluated the proliferation ability between control and Si-MSR LGG cells. *p < 0.05, **p < 0.01, ***p < 0.001. MSR1: macrophage scavenger receptor 1; EMT: epithelial-mesenchymal transition; LGG: lower-grade glioma; siRNA: small interfering RNA.

We compared the mRNA expression level of MSR1 between LGG (TCGA database) and normal brain tissues (GTEx database). As illustrated in Figure 12A, MSR1 was significantly upregulated in LGG samples compared with normal brain tissue. We used siRNA to knockdown the expression of MSR1 and then evaluated the effect of MSR1 on the biofunctions of glioma cells, including migration, invasion, EMT, and proliferation. Compared to Si-NC transfected cells, the MSR1 expression in SHG44 and HS683 cells transfected with Si-MSR1 was significantly downregulated (Figure 12B). Afterward, transwell migration and invasion assays were conducted to test glioma cell migration and invasion abilities. Cell migration (Figure 12C) and invasion abilities (Figure 12D) were dramatically decreased after MSR1 knockdown in glioma SHG44 and HS683 cells. Considering that E-cadherin was not expressed in the conventional glioma cell lines except the SF767 cell, we detected the protein expression of two common EMT markers (Vimentin and ZO-1) in SHG44 and HS683 cells to investigate the EMT process in glioma. We observed significantly decreased expression levels of vimentin as well as dramatically increased expression levels of ZO-1 after the MRS1 knockdown in SHG44 and HS683 cells (Figure 12E). The CCK8 experiment showed a markedly reduced cell proliferation after MSR1 down-regulation in SHG44 and HS683 cells (Figure 12F). These findings indicated that MSR1 was an oncogene associated with the migration, invasion, EMT, and proliferation of glioma cells.

## Discussion

Recent studies suggested that inflammation plays a pivotal role in glioma initiation, progression, and prognosis (Yeung et al., 2013; Li et al., 2014; Michelson et al., 2016). For instance, TNF-α is a master regulator of the inflammatory response, overexpressed and secreted in the neoplasm microenvironment (Ramaswamy et al., 2019). TNF-α strengthens glioma proliferation, migration, and therapy resistance by activating NF-κB signaling (Guo et al., 2017; Geeviman et al., 2018). These processes are related to gene expression changes, including alterations in inflammation genes. Some predictive signatures linked to autophagy, immunity, ferroptosis, and pyroptosis have been constructed in gliomas. However, the relationship between genomic biomarkers based on inflammatory response and the clinical outcome of LGG remains an outstanding question. The present study found that the DE-IRGs can classify LGG patients into two subtypes, which exhibited significant differences in clinical and molecular features. A prognostic signature integrating nine IRRGs was constructed in the TCGA cohort and validated in the CGGA cohort. LGG patients were divided into “high” or “low” risk subgroups using the median risk score as a cutoff value. We found that the high-risk group was significantly linked to older age, higher tumor grade, the wild status of IDH1, 1p/19q non-codeletion, unmethylated status of the MGMT promoter, and shorter survival. The multivariable Cox regression analysis identified the inflammatory signature as an independent prognostic marker for LGG in the training and validation cohorts, similar to the classical prognostic factors, such as tumor grade and 1p/19q codeletion.

To further elucidate the role of these nine genes in LGG, we analyzed their main molecular functions. The prognostic signature constructed in the present study consisted of nine IRRGs (PLA2G2A, MSR1, ABCC3, COL8A1, SVOP, CXCL10, NAPSB, SAA1, and CHI3L1), and all prognostic genes were risky factors, with the single exception of SVOP. Secreted PLA2, PLA2G2A-encoded protein, was discovered to induce proliferation in astrocytoma through the epidermal growth factor receptor (EGFR), contributing to worsening the prognosis of a tumor in an inflammatory microenvironment. (Hernández et al., 2010). MSR1, encoding the class A macrophage scavenger receptors, was reported to be influential in cancer progression and metastasis *in vitro* and *in vivo* (Shigeoka et al., 2015) and to be a marker of tumor-infiltrated macrophages within the tumor microenvironment in glioma (Miyasato et al., 2017). MSR1 could also be a target for intensifying the current anti-glioma therapy (Sørensen and Kristensen, 2022). Our *in vitro* experiments also confirmed that MSR1 was involved in the migration, invasion, EMT, and proliferation of glioma cells. Pessina et al. reported that ABCC3 could protect NK cells from chemotherapy in a murine model with malignant glioma and had significant clinical implications for patients treated with chemo-immunotherapy (Pessina et al., 2016). A high expression of COL8A1 correlated with the poor overall survival in GBM (Jiang et al., 2021). CXCL10 showed tumor-promoting properties and the manifestation of chemokine receptor/ligand pair CXCR3/CXCL10 had an essential role in the proliferation of glioma (Maru et al., 2008). SAA1, a major acute-phase protein, is highly expressed in response to inflammation and tissue injury, and SAA1’s high expression is significantly linked to poor differentiation of tumor cells (Sudo et al., 2021). A previous study showed that increased mRNA level of CHI3L1 could be associated with poor patient survival for glioblastoma and lower-grade astrocytoma tumors (Steponaitis et al., 2016). These studies were consistent with our results that PLA2G2A, MSR1, ABCC3, COL8A1, CXCL10, SAA1, and CHI3L1 were risky factors. Few articles study the relationship between glioma prognosis and SVOP or NAPSB, which need to be further studied.

The malignant proliferation of glioma cells breaks the normal homeostasis within normal brain cells, supporting the formation of the tumor immune microenvironment characterized by an immune-inflammatory response. A previous study has analyzed the prognostic value as well as the effects on the immune microenvironment of neuregulin family members in glioma (Zhao et al., 2021). Another research explored the predictive value of mutated genes and assessed their immune infiltration in LGG (Lin et al., 2021). Our analysis also studied the correlation between risk score and immune status after establishing the IRRG signature. We found that the enrichment scores of immune cells and immune-related pathways based on ssGSEA were significantly higher in the high-risk group than in the low-risk group, except for NK cells. A previous study indicated that immune cells could enter the central nervous system as a result of tissue injury or inflammation caused by malignant gliomas (Rascher et al., 2002), which was consistent with our findings and partially explained why patients in the high-risk group with more immune cells had a poorer prognosis than patients in the low-risk group. In addition, the ESTIMATE algorithm suggested that the risk score was positively correlated with the estimate score, immune score, and stromal score but negatively with tumor purity, further suggesting that inflammatory response and immune regulation were imbalanced in the high-risk group and that the risk signature could serve as a novel indicator of the immune inflammatory response in LGG. Moreover, we also predicted the immunotherapy response by the inflammation-related signature and the result showed that a higher expression of PD-1, PD-L1, and CTLA4, which have been demonstrated to be high-value targets in regulating immunosuppression in glioma (Wainwright et al., 2014), in the high-risk prognostic group than low-risk prognostic group, suggesting that the patients in the high-risk group might be more suitable for immunotherapy. By blockade of PD-1/PD-L1 and CTLA4, immune checkpoint inhibitors can mitigate the suppressive effect on immune cells within the tumor immune microenvironment and enhance endogenous anti-tumor immunity.

Inflammatory cytokines have been demonstrated to correlate oncogenic signaling with the production and maintenance of cancer stem cells (CSCs) (Markopoulos et al., 2019). The inflammatory microenvironment promotes angiogenesis and tumor growth and generates a powerful niche supporting CSCs (Stallone et al., 2019). A previous study suggested that clinical stages and pathology features in glioma were positively correlated with mDNAsi while being negatively correlated with mRNAsi. (Malta et al., 2018), which was in accordance with our results. The correlation indicated that the risk of the prognostic signature might be strongly linked with the activity of CSCs and demonstrated the effectiveness of the predictive model indirectly from another aspect. Several recent pieces of research have revealed a link between m6A modifications and inflammation-related genes in tumor microenvironments (Hou et al., 2019; Rong et al., 2019; Ding et al., 2020; Zhang et al., 2020). The low expression of FTO, the first m6A RNA-demethylase identified, was associated with poor outcomes in glioma (Xu et al., 2020), in line with our correlation analysis, which implied that m6A RNA modification might be directly or indirectly connected to inflammation in LGG, providing a certain reference value for our in-depth research or other people's research in the future.

Analysis of the NCI-60 cell line set in the CellMiner database indicated that the increased levels of the ABCC3 gene and SAA1 gene in the signature were positively correlated to drug resistance, such as tamoxifen and arsenic trioxide. It has been confirmed that tamoxifen exhibited excellent therapeutic effects on temozolomide-insensitive glioma cells (He et al., 2015). Arsenic trioxide, an already FDA-approved drug for leukemia treatment, has recently been reported as a novel anti-glioma drug by regulation of apoptosis and autophagy (Fang and Zhang, 2020). Our results also confirmed that ABCC3 attenuated the chemosensitivity of glioma cells to arsenic trioxide. Hence, LGG patients with low expressions of ABCC3 or SAA1 might benefit from arsenic trioxide or tamoxifen compared with patients with high expressions of ABCC3 or SAA1. These relationships made us hypothesize that the mechanisms of arsenic trioxide and tamoxifen in glioma treatment might also be involved in inducing an inflammation microenvironment. Thus, analyzing the correlation of cancer cell sensitivity to chemotherapy suggested that the inflammatory response subtype-related prognostic molecules are promising for anti-tumor drug development to improve the survival of LGG, and the signature could be a potential indicator for targeted therapy.

## Conclusion

In summary, the already established prognostic signature based on nine IRRGs not only forecasted the prognosis of LGG patients but also reflected the immune characteristics, tumor stemness, m6A mRNA status, and cancer chemoresistance of different risk groups. Our study provides a new constraint from inflammatory response in the development and progression of LGG. However, the peculiar underlying mechanisms for the relationship between the inflammation response and cancer immunity remain unclear and need further investigation.

## Data Availability

The datasets presented in this study can be found in online repositories. The names of the repository/repositories and accession number(s) can be found in the article/Supplementary Material.
