# Supplementary figures and images for: Integrated analysis of inflammatory response subtype-related signature to predict clinical outcomes, immune status and drug targets in lower-grade glioma
Source: Front Pharmacol. 2022 Aug 26;13:914667. doi: 10.3389/fphar.2022.914667 (PMC9459010; doi:10.3389/fphar.2022.914667)

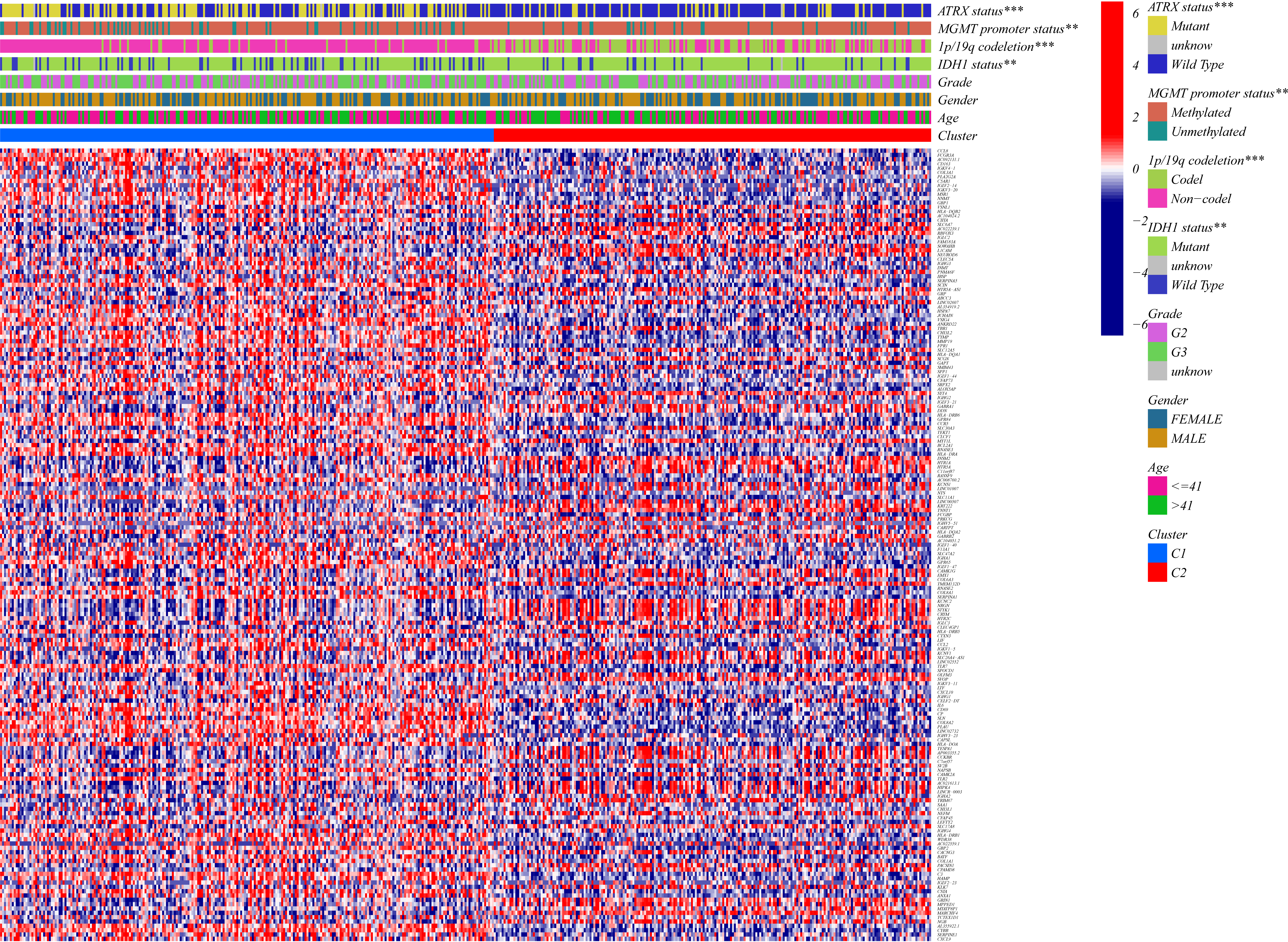

Supplement: Supplementary file 3 [file Image1.TIF]
